# Heavy or light: is muscle fiber growth load-specific? A systematic review and meta‐regression

**DOI:** 10.3389/fphys.2026.1953601

**Published:** 2026-10-06

**Authors:** Dorian Varovic, Stian Larsen, Jozo Grgic

**Affiliations:** 1 Faculty of Kinesiology, University of Zagreb, Zagreb, Croatia; 2 Department of Sports Science and Physical Education, Nord University, Levanger, Norway; 3 Academy for Personal Training, Fredrikstad, Norway; 4 Department of Sports Science and Physical Education, Faculty of Education, The Chinese University of Hong Kong, Hong Kong, Hong Kong SAR, China

**Keywords:** biopsy, muscle hypertrophy, physiology, systematic review, training intensity

## Abstract

**Introduction:**

Training load, expressed as a percentage of one-repetition maximum (%1RM), is an important variable in resistance training. Prior studies comparing low- and high-load training on muscle fiber hypertrophy reported conflicting findings, and the load-specific relationship has not been analyzed in a continuous manner. This systematic review and meta-regression explored the relationship between training load and muscle fiber hypertrophy.

**Methods:**

A systematic search was conducted in PubMed/MEDLINE, Scopus, and SPORTDiscus databases. Standardized mean changes (SMC) were calculated and analyzed using multilevel meta-regressions with cluster-robust variance estimation, with exploratory sensitivity and interacting moderator analyses. Eight studies involving 195 participants were included.

**Results:**

In the fiber-type specific model, pairwise contrast estimates were negative at low loads (20–30% 1RM: −0.147 to −0.088 SMC) and positive at higher loads (60–90% 1RM: 0.089–0.265 SMC). However, the 95% confidence intervals included zero for all pairwise contrasts.

**Conclusion:**

The observed model estimates suggested a potential fiber-type specific pattern across loading ranges. However, these findings should be considered preliminary and interpreted with caution given the small number of included studies and wide 95% confidence and prediction intervals, indicating a degree of uncertainty and imprecision.

## 1 Introduction

Skeletal muscle fibers are commonly categorized according to their predominant myosin heavy chain (MyHC) isoform expression into type I and type II fibers (Plotkin et al., 2021; Blemker et al., 2024). Type I fibers exhibit slower contraction speeds and high fatigue resistance. Type IIa fibers generate force more rapidly than type I fibers but fatigue sooner, while type IIx fibers produce the fastest contractions yet are highly susceptible to fatigue (Schiaffino and Reggiani, 2011; Blemker et al., 2024). The size of skeletal muscle fibers displays remarkable plasticity and can substantially change in response to stimuli such as resistance training (Campos et al., 2002; Schuenke et al., 2012).

Training load is one of the key variables in resistance training programming and is generally expressed as a percentage of one-repetition maximum (1RM) (Schoenfeld et al., 2021a). Current guidelines suggest that muscle hypertrophy can be achieved across a wide spectrum of loads, provided that sets are performed within close proximities to momentary muscular failure (Schoenfeld et al., 2016, 2017, 2021b; Lopez et al., 2021; Carvalho et al., 2022). This adaptation is largely explained by the size principle of motor unit recruitment, whereby decreases in proximity to failure tend to result in increased motor unit recruitment of both lower- and higher-threshold motor units (Henneman, 1985; Robinson et al., 2024). Still, it should be noted that these findings are specific to studies using assessment tools such as ultrasound or magnetic resonance imaging, which do not provide insight into the adaptations occurring at the muscle fiber level (Haun et al., 2019). Given the differences in contractile speed, force capacity, and fatigue resistance among fiber types, some researchers propose that low-load training may preferentially stimulate type I fiber hypertrophy, whereas high-load training could favor growth of type II fibers (Grgic and Schoenfeld, 2018; Grgic et al., 2018; Schoenfeld et al., 2023). Meta-analyses comparing low- and high-load training have examined this hypothesis, yet findings remain largely inconclusive (i.e., no significant differences in pairwise comparisons) (Grgic, 2020; Refalo et al., 2021).

A limitation in previous meta-analyses (Grgic, 2020; Refalo et al., 2021) is that they explored this topic using binary classifications (low- versus high-load), even though load is inherently continuous. Modeling load as a continuous variable allows for a more nuanced characterization of both the magnitude and shape (linear or non-linear) of the load–specific relationship (Royston et al., 2006; Naggara et al., 2011; Fisher et al., 2022; Robinson et al., 2024; Pelland et al., 2026). Another important consideration is that previous analyses also accounted for correlated effects within a study by using averages, which may result in the loss of information. Multilevel modeling approaches, with appropriate nesting structures, may result in greater statistical power and better account for the dependency and variance among observations within and between studies (Harrer et al., 2021). Additionally, new studies (Cumming et al., 2025; Lees et al., 2025) have been published since the most recent meta-analyses, underscoring the need for an updated synthesis. Therefore, using multilevel meta-regressions, we aimed to explore: (a) the relationship between training load and muscle fiber hypertrophy; and (b) the impact of potential moderators on this relationship.

## 2 Materials and methods

### 2.1 Search strategy

This systematic review and exploratory meta-regression were conducted in accordance with the Preferred Reporting Items for Systematic Reviews and Meta-Analyses guidelines (Page et al., 2021). This study was not prospectively pre-registered. The original database searches were restricted to studies published between January 2020 and 27 February 2026 because a previous meta-analysis conducted by one of the authors of the present review had searched the literature up to January 2020 (Grgic, 2020). All studies included in the latest meta-analyses on the topic were included in the present article as well (*n* = 5). Primary searches were performed in PubMed/MEDLINE, Scopus, and SPORTDiscus databases using the following search syntax: (“high-load” OR “high load” OR “low load” OR “low-load” OR “high repetition” OR “low repetition” OR “higher-repetition” OR “lower repetition” OR “exercise load” OR “training load” OR “traditional muscular endurance” OR “traditional muscular strength”) AND (“cross sectional area” OR “CSA” OR “muscle fiber” OR “muscle fiber” OR “type I” OR “type II” OR “type IIa” OR “type IIx” OR “muscle biopsy” OR “muscle biopsies” OR “hypertrophy” OR “muscle size” OR “muscle growth”). Secondary searches were performed by screening Open Access Theses and Dissertations, a database indexing master’s and doctoral theses. In addition, reference lists of the included studies (i.e., backward citation tracking) and articles citing the included studies (i.e., forward citation tracking) were examined through Google Scholar. Finally, reference lists of the existing review articles (Grgic and Schoenfeld, 2018; Grgic, 2020; Refalo et al., 2021) were screened for relevant studies. The primary and secondary searches were conducted by DV and completed on 2 March 2026. All identified studies were imported into Zotero reference management software (version 7.0.32). After duplicates were removed, DV and SL screened titles and abstracts to determine initial eligibility. Studies meeting the inclusion criteria were moved to the full-text screening stage. DV and SL screened the full texts to determine final inclusion. Disagreements were resolved through mutual discussion or with a third author.

In August, an updated search of the databases, without the temporal restriction, was performed by DV and JG. Records identified through the updated searches were independently screened for eligibility. No additional studies meeting the inclusion criteria were identified; therefore, the final set of eight included studies remained unchanged.

### 2.2 Inclusion criteria

To be included in this meta-analysis, studies had to: (1) be written in English and published in a peer-reviewed journal, as a preprint, or as a master’s or doctoral thesis; (2) involve adults (<60 years) with no known medical conditions or injuries; (3) employ a resistance training intervention utilizing a randomized experimental design (within- or between-group) lasting a minimum of 4 weeks; (4) compare at least two groups or conditions differing in training load (e.g., %1RM) throughout the training intervention; and (5) include pre- and post-intervention measurements of muscle hypertrophy at the muscle fiber level (fiber cross-sectional area via biopsy). If these criteria were not satisfied, studies were excluded from consideration.

### 2.3 Data extraction

The following data were extracted by the lead author from the included studies into a predefined coding spreadsheet: (1) study author, year of publication, and design; (2) participants’ age, body mass, height, sex, and training experience; (3) intervention groups/conditions (including whether they were volume equated and performed sets to failure); (4) resistance training protocols (i.e., number of sets, exercises performed, load used); (5) intervention duration; (6) muscle group analyzed; (7) specific outcome (e.g., cross-sectional area) and method of assessment (e.g., mATPase histochemistry or MyHC antibody); (8) pre- and post-intervention data for all relevant outcomes [e.g., mean ± standard deviation (SD)]. When the data were not reported in the full text or When the data were not reported in the full text or Supplementary Materials, the lead or corresponding authors of those studies were contacted to request missing data. If the authors did not respond, data were obtained, when possible, using WebPlotDigitizer (version 4.8; Ankit Rohatgi). Another author (JG) independently verified the complete extracted dataset and coding procedures. Any identified discrepancies were resolved through mutual discussion between the authors.

### 2.4 Quality assessment

Study quality was assessed using the Tool for the assEssment of Study qualiTy and reporting in EXercise (TESTEX) scale (Smart et al., 2015). The TESTEX scale is specifically designed to evaluate the methodological quality and reporting of exercise training studies. The scale includes 12 criteria with a maximum score of 15 points, comprising five points for study quality (i.e., eligibility criteria specified, randomization, allocation concealment, baseline group similarity, and assessor blinding) and ten points for study reporting (i.e., outcome measures assessed in ≥85% of participants, adverse events, intention-to-treat analysis, between-group statistical comparisons, reporting of point estimates and variability, monitoring of physical activity outside the intervention, reporting of exercise intensity, energy expenditure and other exercise parameters during the training intervention). DV and SL independently performed the assessments, with any discrepancies resolved through mutual discussion or by the third author.

### 2.5 Training load estimation and fiber type classification

Included studies reported repetition ranges (e.g., 3–5 RM) or loading ranges (e.g., 90-95% 1RM). When an exact %1RM was reported, this value was used directly. When a %1RM range was reported, the midpoint of the prescribed range was used as the representative training load. When only a repetition range (i.e., 3–5 RM) was reported without a corresponding %1RM value, the midpoint of the repetition range was converted to an estimated %1RM using the estimates from a recent meta-regression (Nuzzo et al., 2024). When both repetition and %1RM ranges were reported, the directly reported %1RM range was used. Conversion using the estimates from a meta-regression (Nuzzo et al., 2024) was performed only for one study (Campos et al., 2002). In addition to the primary fiber types (type I and type II), MyHC isoform identification enables further classification into fiber subtypes such as IIa and IIx, as well as hybrid fibers co-expressing multiple isoforms (e.g., type IIa/x). Earlier studies often referred to type IIx fibers as type IIb; however, true type IIb fibers are rarely recognized in adult human skeletal muscle (Blemker et al., 2024). Therefore, for fiber-type specific models, studies reporting type IIb fibers were coded as type IIx.

## 3 Statistical analysis

All statistical analyses were performed using R and RStudio (version 4.5.2; R Core Team, https://www.r-project.org). The extracted dataset, analyses, scripts, and Supplementary Materials are available on Open Science Framework (https://osf.io/wgze9/). Given the goal of these exploratory analyses, our interpretation focused on the effect estimates, 95% confidence intervals (CI), and prediction intervals (PI) (Gardner and Altman, 1986). An arm-based meta-analysis was performed using the *metafor* package (Viechtbauer, 2010). As included studies had multiple groups or conditions and reported effects within these groups or conditions for multiple outcomes, we calculated effect sizes in a nested structure to account for multiple observations reported within a given study. Therefore, multilevel mixed-effects models (Van den Noortgate et al., 2013) with cluster-robust variance estimation (Hedges et al., 2010) were performed with study, group, and observation included as nested random intercepts in the model (i.e., observations were nested within groups that were nested within studies). Variance components were estimated with restricted maximum likelihood (REML), and effect sizes were calculated using inverse-variance weighting to account for the study-level, group-level, and observation-level variance. Effect sizes were calculated as standardized mean change (SMC) using the *escalc* function (Becker, 1988; Viechtbauer, 2010). Specifically, SMC was calculated as the difference between post-intervention and pre-intervention means divided by the pre-intervention SD with an adjustment for small samples (i.e., C).


$$SMC=C(\frac{M_{post\;-}M_{pre}}{SD_{pre}});\;C=1-\frac{3}{4(n-1)-1}$$

The SMC magnitude was interpreted as: trivial (<0.20), small effect (0.20-0.49), moderate (0.50-0.79), and large (≥0.80) effect as per Cohen’s classification (Cohen, 2013). When a study reported standard errors, these were converted to SD. When neither was available but interquartile ranges were reported, SDs were estimated assuming a normal distribution^1^. Interquartile ranges were back-calculated to SDs using the following equation (Jané, 2023):


$$SD=\frac{Q_{3}-Q_{1}}{z_{0.75}-z_{0.25}}$$

One study (Lees et al., 2025) reported change scores for each condition, and reported baseline values for one limb across the sample. To compute SMC, we used the pooled baseline SDs reported per fiber type as the denominator for both conditions. The pre-intervention mean was set to zero and the change score was treated as the post-intervention mean, with the pooled baseline SD as the pre-SD (Viechtbauer, 2010).

None of the included studies reported pre- to post-intervention correlations to determine the variance for calculating effect sizes. However, one study (Cumming et al., 2025) reported individual-level pre- and post-intervention data in figures for each fiber type (type I and type II) within each condition. These data were manually extracted using the WebPlotDigitizer to derive correlation coefficients and imputed for the studies where we were unable to obtain the required data. We did not have data to calculate correlation coefficients for studies specifying fiber subtypes (e.g., type IIa, type IIx). Therefore, correlation coefficients derived from pooled type II fibers (*r* = 0.9) were deemed appropriate and used to determine variances.

To account for a potential non-linear relationship between training load and muscle fiber hypertrophy, candidate functional forms were fit as three-level nested random intercepts models using the *metafor* package with cluster-robust variance estimation and subsequently compared using the *performance* package (Lüdecke et al., 2021). Bayesian information criterion (BIC)-approximated Bayes factor (BF) was used to determine under which model the observed data were most probable. All candidate models were compared with and without study design as a covariate, as study design may partially explain model variance independently of training load. For the overall fiber model, candidate models including only a main effect of load were compared relative to an intercept-only baseline. For the fiber-type specific model, candidate models were compared against a baseline including fiber type as a fixed effect. Both additive (load + fiber type) and interaction (load × fiber type) parameterizations were tested for each functional form. The comparison of these models using 2 x ln(BF) was made according to the Kass & Raftery (Kass and Raftery, 1995) scale where: <0 negative evidence in favor of the candidate model; 0–2 weak; 2–6 positive; 6–10 strong; >10 very strong evidence in favor of the candidate model relative to the intercept models. The model that performed the best was considered the “best fit” and used for all subsequent analyses. Where several competing models performed similarly, the simpler model was preferred on the basis of parsimony. The following functional forms were tested:

1. Linear
2. Linear-log.
3. Restricted cubic spline (2 knots)
4. Quadratic

Models that focused on the relationship between training load and fiber-type specific muscle hypertrophy included the following fixed effects: (1) training load; (2) fiber type; (3) interaction term between training load and fiber type; and (4) study design. A sensitivity model was performed in which type II fiber data were restricted to studies reporting aggregated type II fibers, while the model otherwise retained the same structure as the fiber-type-specific model.

Estimated marginal means and their slopes with 95% CIs and PIs were extracted for the main effect of load, proportionally marginalized over other predictors, using the *emmeans* package (Lenth and Piaskowski, 2017). Estimated marginal means were extracted at prespecified load anchors ranging from 20% to 90% 1RM, representing the observed range of training loads in the dataset. For the fiber-type specific models, pairwise contrasts of estimated marginal means between type I and type II muscle fiber hypertrophy were then computed at each load anchor to characterize load-specific differences between fiber types.

The proportion of variability in effect estimates due to heterogeneity rather than sampling error was estimated using the I^2^ statistic (Nakagawa and Santos, 2012). Thresholds for the interpretation of I^2^ were considered as low (<50%), moderate (50-75%), or high (>75%). Given the use of a multi-level arm-based model, the total I² was partitioned across the three levels of nested random effects (i.e., study, group, observation). Similarly, *τ²* estimates were obtained for each random-effects level, representing the absolute magnitude of heterogeneity at the study, group, and observation levels. Marginal and conditional pseudo R^2^ were calculated to quantify the proportion of variance explained by only the fixed effects alone and the sum of the fixed and random effects, respectively (Nakagawa et al., 2017).

Sensitivity leave-one-out analyses were performed for SMC fiber-type specific models to explore whether any single study may have unduly influenced the model results. Additionally, these analyses allowed us to explore whether model outcomes were impacted by inclusion of a study that did not perform training to muscular failure (Mackey et al., 2011).

Moderator analyses were performed only for the SMC fiber-type specific models with the *metafor* package to investigate the influence of various factors related to study design and participant characteristics. Prior to fitting the models, multicollinearity between moderators of interest was assessed using the variance inflation factors (VIFs). Separate models were fitted for each moderator that maintained the same structure as the best fit model but also included a linear main effect and interaction term between training load and the moderator of interest (i.e., training status, direct sets per week, volume equated studies, study design, rest intervals between sets).

## 4 Results

### 4.1 Search results

During the original search process, the restricted database searches identified 1,004 results. Out of these search results, 26 full texts were assessed for eligibility, and two studies (Cumming et al., 2025; Lees et al., 2025) were included in the review. Secondary searches yielded another 4,527 results, and one additional study (Mackey et al., 2011) was found to satisfy the inclusion criteria. Therefore, in addition to the five studies (Campos et al., 2002; Mitchell et al., 2012; Schuenke et al., 2012; Morton et al., 2016; Lim et al., 2019) from the previous meta-analyses, the total number of analyzed studies was eight (Campos et al., 2002; Mackey et al., 2011; Mitchell et al., 2012; Schuenke et al., 2012; Morton et al., 2016; Lim et al., 2019; Cumming et al., 2025; Lees et al., 2025). The updated searches identified 2,213 results. After removing 921 duplicates, 1,292 titles and abstracts were screened, of which 1,272 were excluded. Twenty full-text articles were assessed for eligibility. Nineteen were excluded because of an unsuitable study design, and one study was excluded because it was a subset of data from an already included study (Morton et al., 2016). No additional eligible studies were identified through the updated searches. Therefore, the final set of eight studies remained unchanged (**Figure 1**).

**Figure 1:**
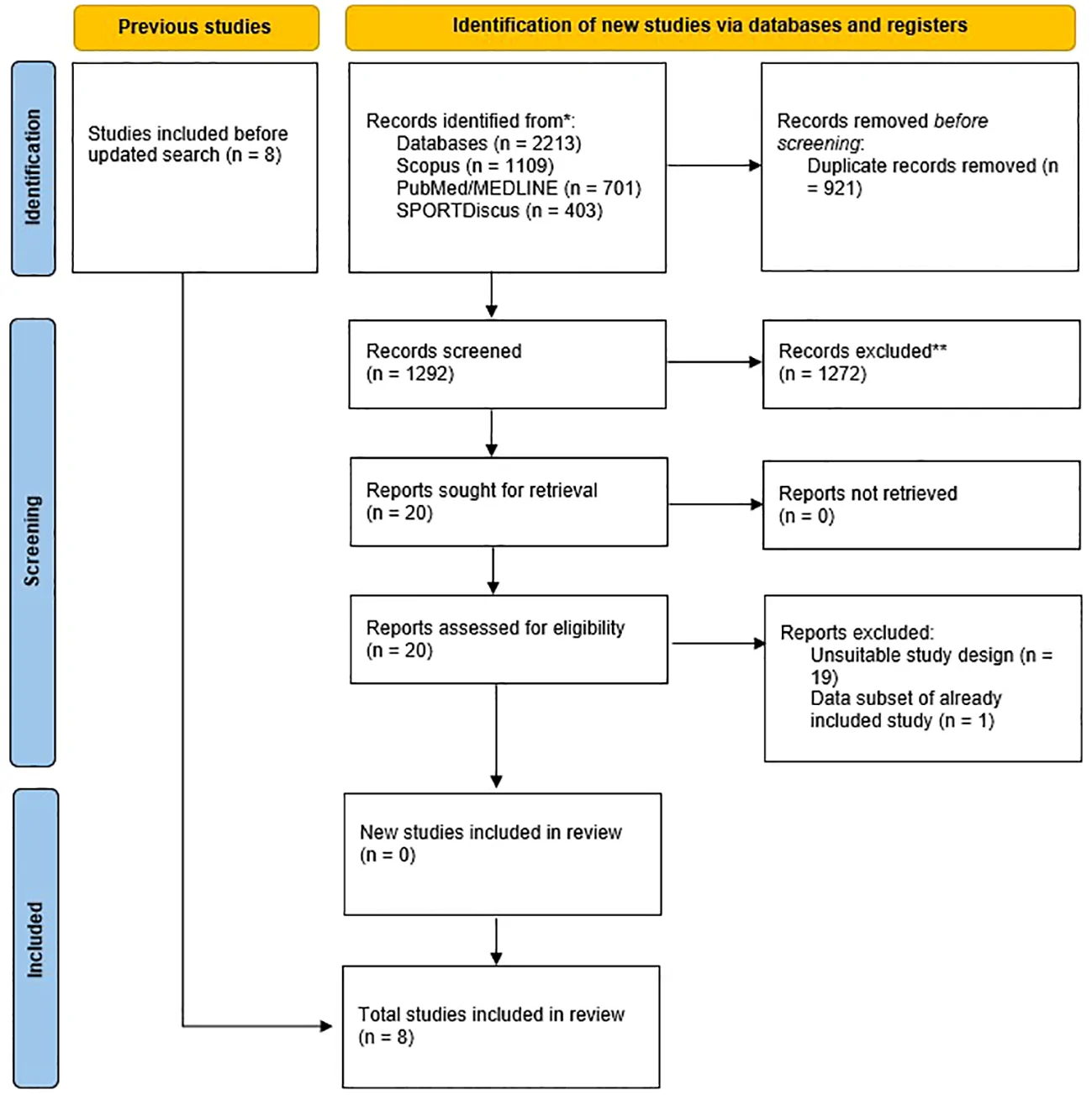
Preferred reporting items for systematic reviews and meta-analyses (PRISMA) flow diagram. The eight studies included before the updated search were identified through the original database and secondary-search procedures described in the methods.

### 4.2 Study characteristics

The pooled number of participants across the included studies was 195. Study samples ranged from 12 to 49 participants (median: 20). All studies included young adult participants (range: 20.8–25.0 years). Six studies (Campos et al., 2002; Mackey et al., 2011; Mitchell et al., 2012; Morton et al., 2016; Lim et al., 2019; Lees et al., 2025) included male-only participants, one study (Cumming et al., 2025) included both male and female participants, and one study (Schuenke et al., 2012) included female-only participants. Training interventions lasted 9.6 ± 2.0 weeks. Three studies (Campos et al., 2002; Mackey et al., 2011; Lees et al., 2025) equated training volume (i.e., volume load) between groups or conditions, whereas five studies (Mitchell et al., 2012; Schuenke et al., 2012; Morton et al., 2016; Lim et al., 2019; Cumming et al., 2025) did not. Four studies (Campos et al., 2002; Schuenke et al., 2012; Morton et al., 2016; Lim et al., 2019) used a between-participant design, whereas four studies (Mackey et al., 2011; Mitchell et al., 2012; Cumming et al., 2025; Lees et al., 2025) used a within-participant design. Two studies (Morton et al., 2016; Cumming et al., 2025) included trained participants, while six studies (Campos et al., 2002; Mackey et al., 2011; Mitchell et al., 2012; Schuenke et al., 2012; Lim et al., 2019; Lees et al., 2025) included untrained participants. Only one study (Mackey et al., 2011) did not have participants perform training to volitional or momentary failure. All studies performed biopsies of the vastus lateralis and measured muscle fiber cross-sectional area. Four studies (Campos et al., 2002; Mackey et al., 2011; Mitchell et al., 2012; Schuenke et al., 2012) used mATPase histochemistry, and four studies (Morton et al., 2016; Lim et al., 2019; Cumming et al., 2025; Lees et al., 2025) used MyHC antibodies. Seven studies (Campos et al., 2002; Mackey et al., 2011; Mitchell et al., 2012; Schuenke et al., 2012; Lim et al., 2019; Cumming et al., 2025; Lees et al., 2025) involved lower-body exercises, whereas one study (Morton et al., 2016) employed a full-body resistance training routine (**Table 1**).

**Table 1:** Summary of the studies included in the systematic review.

Study	Participants	Study design	RT groups/conditions	Intervention duration (sessions/week)	Exercise(s) performed	Volume equated/performed to failure?	Method of assessment	TESTEX scale (/15)
Campos et al. (2002)	Physically active young males (*n* = 27)	Between-groups	LR: 4 sets x 3–5 RM; INTR: 3 sets x 9–11 RM; HR: 2 sets x 20–28 RM;	8 weeks (2/week first 4 weeks; 3/week remaining 4 weeks)	Leg press, squat, knee extension	Yes/Yes	mATPase histochemistry	7
Cumming et al. (2025)	Resistance-trained young males (*n* = 11) and females (*n* = 3)	Within-groups	HL: 3 sets x 3–5 repetitions; LL: 3 sets x 20–25 repetitions	9 weeks (2/week)	Inclined leg press, knee extension	No/Yes	MyHC antibody	11
Lees et al. (2025)	Untrained young males (*n* = 20)	Within-groups	HL: 3 sets x 8–12 repetitions; LL: 3 sets x 20–25 repetitions	10 weeks (3/week)	Unilateral biceps curl, knee extension	Yes/Yes	MyHC antibody	9
Lim et al. (2019)	Untrained young males (*n* = 21)	Between-groups	80FAIL: 3 sets at 80% 1RM 30FAIL: 3 sets at 30% 1RM	10 weeks (3/week)	Leg press, knee extension, leg curl	No/Yes	MyHC antibody	8
Mackey et al. (2011)	Untrained young males (*n* = 12)	Within-groups	HL: 10 sets x 8 repetitions at 70% 1RM LL: 10 sets x 36 repetitions at 15.5% 1RM	12 weeks (3/week)	Knee extension	Yes/No	mATPase histochemistry	8
Mitchell et al. (2012)	Physically active young males (*n* = 18)	Within-groups	80%-3 sets: 3 sets at 80% 1RM 30%-3 sets: 3 sets at 30% 1RM	10 weeks (3/week)	Knee extension	Yes/Yes	mATPase histochemistry	6
Morton et al. (2016)	Resistance-trained young males (*n* = 49)	Between-groups	HR: 3 sets x 20–25 repetitions; LR: 3 sets x 8–12 repetitions;	12 weeks (4/week)	Seated row, bench press, shoulder press, front plank, bicep curls, triceps extension, wide grip pull-downs, leg press, knee extension, and knee flexion.	No/Yes	MyHC antibody	10
Schuenke et al. (2012)	Untrained young females (*n* = 34)	Between-groups	TS: 3 sets x 6–10 RM with 1–2 s concentric/1–2 s eccentric phase duration; TE: 3 sets x 20–30 RM with 1-2s concentric/1-2s eccentric phase duration;	6 weeks (2/week first week; 3/week remaining 5 weeks)	Leg press, squat, knee extension	Yes/Yes	mATPase histochemistry	7

LR: low repetition; INTR: intermediate repetition; HR: high repetition; RM: repetition maximum; HL: high-load; LL: low-load; %1RM: percentage one-repetition maximum; MyHC: myosin heavy chain; Note: all studies performed biopsies of the vastus lateralis muscle.

### 4.3 Quality assessment

Study-level TESTEX scores can be found in the **Supplementary Materials** (https://osf.io/43ucs). The mode score on the TESTEX scale was 7/15 (range: 6–11/15). The mode study quality score was 2/5 (range: 1–3/5). Studies were commonly downgraded for not clearly specifying eligibility criteria for inclusion (Campos et al., 2002; Mackey et al., 2011; Mitchell et al., 2012; Lees et al., 2025), and for not reporting blinding of assessors for at least one outcome (Campos et al., 2002; Mitchell et al., 2012; Schuenke et al., 2012). None of the included studies reported methods of randomization or allocation concealment. The mode study reporting score was 5/10 (range: 5–8/10). Several studies were downgraded for not reporting drop-out and adherence information or not including reasons for withdrawal (Mitchell et al., 2012; Schuenke et al., 2012; Lim et al., 2019).

### 4.4 Primary analyses

#### 4.4.1 Overall model

The intercept model with study design added as a predictor showed the strongest evidence over the intercept-only baseline (2×ln(BF) = 6.62). Adding load as a predictor did not provide additional evidence beyond study design. The estimated marginal mean was 0.489 SMC (95% CI: 0.297, 0.682; 95% PI: −0.364, 1.343), averaged proportionally over study design. The fixed effects explained 36.1% of the total variance (R^2marginal^) while 70.7% of the total variance was explained by both fixed and random effects (R^2conditional^). Total heterogeneity not attributable to sampling error was moderate and primarily occurred at the group level (I^2total^ = 69.1%; τ^2group^ = 0.06).

#### 4.4.2 Fiber-type specific model

Model comparisons revealed that linear-log (2×ln(BF) = 7.16) and linear (2×ln(BF) = 7.12) models with study design added as a predictor performed similarly. Therefore, the linear model was used for the subsequent analyses on the basis of parsimony (**Figure 2**). The marginal slope of training load for type II fibers was β = 0.054 (95% CI: −0.038, 0.145; 95% PI: −0.729, 0.836). The marginal slope of training load for type I fibers was β = −0.005 (95% CI: −0.082, 0.072; 95% PI: −0.786, 0.776). Pairwise contrasts of estimated marginal means were negative at low loads (20% 1RM: −0.147 SMC [95% CI: −0.345, 0.052]; 30% 1RM: −0.088 SMC [95% CI: −0.252, 0.076]) and compatible with negligible effects at moderate loads (40–50% 1RM). Above 50% 1RM, these contrasts were 0.089 (95% CI: −0.076, 0.253), 0.147 (95% CI: −0.052, 0.347), 0.206 (95% CI: −0.037, 0.449), 0.265 (95% CI: −0.026, 0.556) for 60%, 70%, 80%, and 90% 1RM, respectively. Fixed effects explained 41.5% of the total variance (R^2marginal^) while 73.4% of the total variance was explained by both fixed and random effects (R^2conditional^). Residual heterogeneity was moderate and primarily occurred at the group level (I^2total^ = 69.4%; τ^2group^ = 0.06).

**Figure 2:**
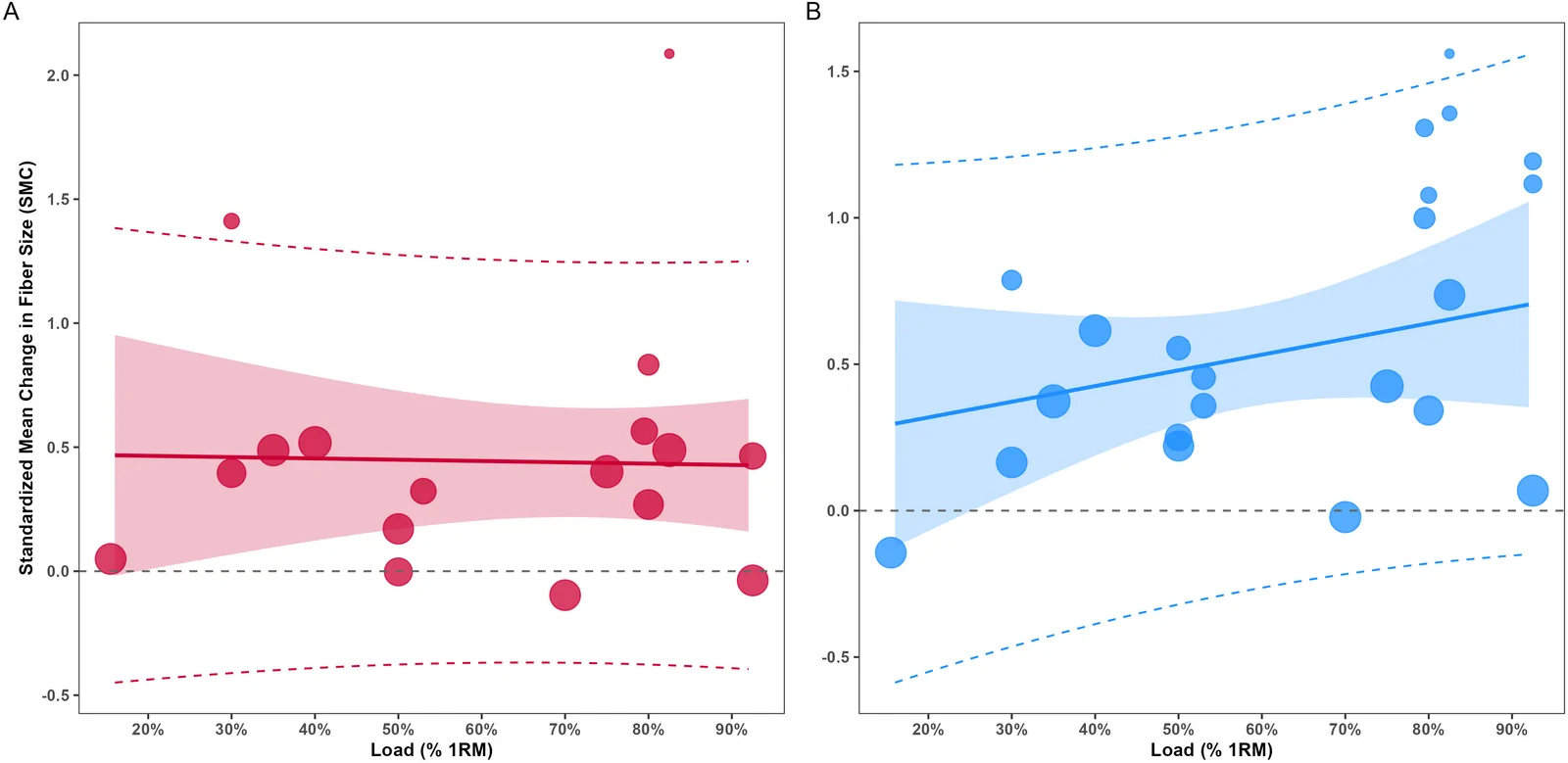
Load-specific relationship between training load and fiber-type specific hypertrophy. Data are presented as estimated marginal means (solid line) with 95% confidence intervals (shaded band) and prediction intervals (dashed lines) after adjusting for study design. Panel **(A)** represents type I fibers and Panel **(B)** represents type II fibers. Circles represent the effect size of each observation, with circle size reflecting inverse variance weighting. Estimated marginal means are presented on the original % of 1 repetition maximum (RM) scale.

### 4.5 Secondary analyses

#### 4.5.1 Aggregated type II fiber model

The aggregated type II fiber sensitivity model retained the same linear functional form as the fiber-specific model (**Figure 3**). The marginal slope of training load for type II fibers was β = 0.019 (95% CI: −0.016, 0.055; 95% PI: −0.680, 0.718), and for type I fibers it was β = −0.017 (95% CI: −0.052, 0.019; 95% PI: −0.716, 0.682). Pairwise contrasts of estimated marginal means were negative at low loads (20% 1RM: −0.108 SMC [95% CI: −0.319, 0.103]; 30% 1RM: −0.073 SMC [95% CI: −0.254, 0.109]). Pairwise contrasts of estimated marginal means were compatible with negligible effects at 40 to 50% 1RM. Above 50% 1RM, these contrasts were 0.035 (95% CI: −0.093, 0.162), 0.070 (95% CI: −0.059, 0.200), 0.106 (95% CI: −0.037, 0.249), 0.142 (95% CI: −0.023, 0.306) for 60%, 70%, 80%, and 90% 1RM, respectively. Fixed effects of the model explained about a quarter of the variance (R^2marginal^ = 27.7%; R^2conditional^ = 66.4%). Following pooling at the observation level, residual heterogeneity was moderate and now attributable to the study level (I^2total^ = 67.9%; τ^2study^ = 0.05).

**Figure 3:**
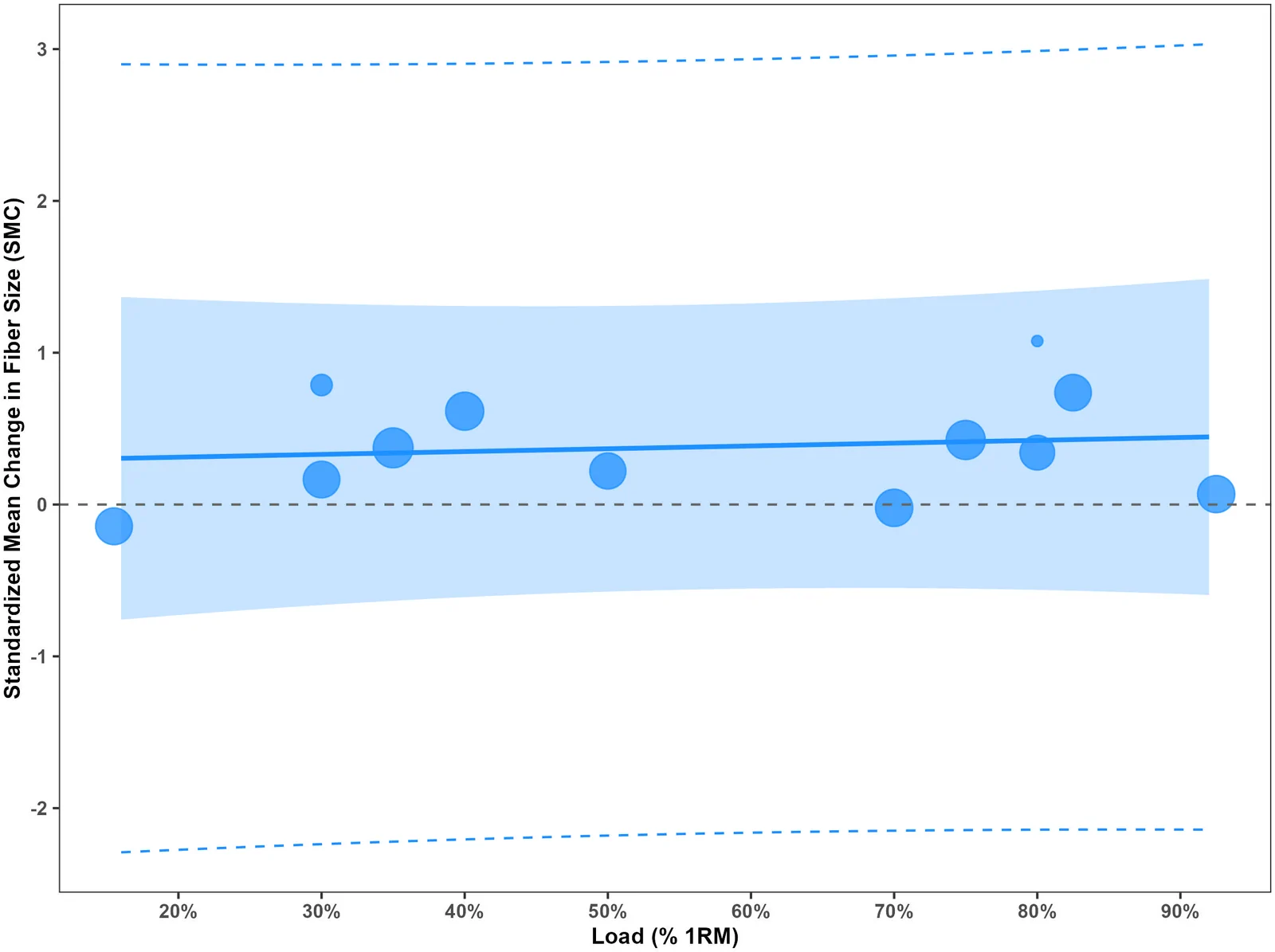
Load-specific relationship between training load and muscle fiber hypertrophy for the aggregated type II fiber sensitivity model. Only data from studies reporting aggregated type II fiber outcomes are presented. Data are presented as estimated marginal means (solid line) with 95% confidence intervals (shaded band) and prediction intervals (dashed lines) after adjusting for study design. Circles represent the effect size of each observation, with circle size reflecting inverse variance weighting. Estimated marginal means are presented on the original % of 1 repetition maximum (RM) scale.

#### 4.5.2 Leave-one-out analysis

Leave-one-out analyses suggested that the load × fiber type interaction was stable in directionality across all eight iterations, with estimates ranging from 0.043 to 0.069. The 95% CIs excluded zero in two of the eight iterations. Removal of Campos et al. (2002) resulted in the smallest interaction estimate [β = 0.043 (95% CI: 0.005, 0.081)], while removal of Mackey et al. (2011) resulted in the interaction estimate of β = 0.059 (95% CI: −0.017, 0.134). Pairwise contrasts at load anchors remained directionally consistent with the primary model across all iterations. Full leave-one-out summaries including marginal slopes, contrasts and estimated marginal means at load anchors can be found here: https://osf.io/b9wat.

### 4.6 Interacting moderator analysis

The estimated marginal slope of training load was β = 0.060 (95% CI: −0.028, 0.149) in volume-equated studies and β = 0.048 (95% CI: −0.092, 0.188) in non-volume-equated studies for type II fibers. Both estimates were compatible with negligible and positive effects. For type I fibers, marginal slopes were negligible in both volume-equated (β = 0.001 [95% CI: −0.045, 0.048]) and non-volume-equated studies [β = −0.011 (95% CI: −0.151, 0.13)]. Data visualization from all moderator analyses for the effects of training load can be found in the **Supplementary Materials** (https://osf.io/dbx56). Due to a limited number of included studies (*k* = 8), we did not formally assess the presence of publication bias or small-study effects.

## 5 Discussion

The present article explored resistance training load-specific effects on muscle fiber hypertrophy. When both fiber types were combined in the model, there was insufficient evidence to determine a meaningful relationship between training load and muscle fiber hypertrophy. However, our fiber-type specific model was compatible with a possible differential fiber-type response across loading ranges. Specifically, at low loads (20-30% 1RM), the model estimates were compatible with greater type I muscle fiber growth. In contrast, as training load increased, the overall model-derived pattern indicated a tendency for greater hypertrophy in type II fibers. However, these findings should be interpreted with caution due to the scarcity of available data, the small number of included studies and the wide 95% CIs and PIs associated with the estimates, many of which included zero.

When using whole muscle tools to assess hypertrophy (i.e., magnetic resonance imaging), previous research has shown that both low load and high load training, if performed to muscular failure, may induce similar muscle growth (Schoenfeld et al., 2017; Refalo et al., 2021). These findings are consistent with the lack of a clear load-specific relationship when both fiber types are pooled in a single model. Indeed, across all analyses, the estimated marginal slopes for type II fibers were compatible with negligible or positive effects, whereas those for type I fibers were compatible with negligible or negative effects. Therefore, when both muscle fiber types are pooled, it may be that the overall load-specific relationship was influenced by the opposing directionality of the observed type I and type II fiber effects.

The fiber-type specific point estimate for the load × fiber type interaction was positive, suggesting that increases in training load may be associated with relatively greater hypertrophy in type II compared to type I fibers. To aid interpretation, we focused on estimated marginal slopes across a 10 percentage-point increase in load around the mean and pairwise contrasts at meaningful load anchors. Overall, the estimated marginal slopes were β = 0.054 (95% CI: −0.038, 0.145) for type II fibers and β = −0.005 (95% CI: −0.082, 0.072) for type I fibers. The 95% CIs included zero for both estimates, indicating substantial uncertainty regarding the direction and magnitude of these effects. Pairwise contrast estimates indicated a tendency for greater type I than type II fiber hypertrophy at low loads (20–30% 1RM), although the 95% CIs included zero. At moderate loads (40–50% 1RM), the estimated hypertrophy responses appeared similar between fiber types. Above 50% 1RM, pairwise contrast estimates were positive in direction, progressively increasing from 0.089 SMC at 60% 1RM to 0.265 SMC at 90% 1RM. However, the 95% CIs included zero for all pairwise contrasts, indicating uncertainty and imprecision. Therefore, these findings should be interpreted with caution.

Three studies (Campos et al., 2002; Schuenke et al., 2012; Lees et al., 2025) reported subtypes of type II fibers (i.e., IIa, IIa/x, IIx). Including these fiber subtypes alongside aggregated type II fiber effects in our fiber-type specific model may have introduced additional group-level variability due to the small number of these fiber subtypes observed across biopsy samples. For example, Mackey et al. (2011) and Lees et al. (2025) note that the prevalence of type IIx and type IIa/x fibers is generally low, and that in their study, some biopsy samples contained few or none of these fiber subtypes. However, in the aggregated type II fiber sensitivity model, marginal slopes for type I (β = −0.017 per 10% 1RM) and type II fibers (β = 0.019 per 10% 1RM) were attenuated, although directionally consistent with the fiber-type specific model (type I fibers: β = −0.005; type II fibers: β = 0.054). This suggests that the primary finding is likely not an artifact of including these fiber subtypes within the model. Restricting the type II data to aggregated outcomes resulted in residual heterogeneity shifting from the group to the study level, suggesting that subtype inclusion in the primary model may have contributed to additional unexplained variance at the group level. As a part of the secondary analyses, removal of the only study (Mackey et al., 2011) in which participants did not perform training to failure resulted in a load × fiber type interaction estimate consistent with the removal of other studies in our leave-one-out analyses (β = 0.059 [95% CI: −0.017, 0.134]). This would suggest that our primary findings were not unduly influenced by this study (Mackey et al., 2011). Nevertheless, future research is needed to explore the overall influence of different proximities to failure with low and high loads.

In subgroup analyses, we explored the influence of other resistance training variables on our findings. Due to the small number of studies, the majority of these analyses were coupled with wide and overlapping 95% CIs. For example, the marginal slope of load for type II fibers was compatible with positive effects in both volume-equated [β = 0.060 (95% CI: −0.028, 0.149)] and non-equated studies [β = 0.048 (95% CI: −0.092, 0.188)]. The 95% CIs included zero in both volume-equated and non-equated studies. However, as mentioned, these analyses were based on very small samples (volume-equated *k* = 3; non-equated *k* = 5), which holds for all moderator analyses. Therefore, these findings should be viewed as hypothesis-generating and warrant additional studies specifically designed to explore the influence of these moderators.

### 5.1 Strengths and limitations

The present study has several strengths. This is the first meta-regression to characterize the relationship between training load and muscle fiber hypertrophy in a continuous manner. We used arm-based multilevel meta-regression models to appropriately account for the dependency and variance among observations within and between studies. In addition, we performed a series of sensitivity analyses to assess the robustness of our findings. Finally, we explored the potential influence of other programming variables on the training load and muscle fiber hypertrophy relationship, primarily as a means of generating hypotheses and informing future research on the topic.

Despite these strengths, several limitations should be considered when interpreting the findings of the present systematic review and meta-analysis. The majority of included studies involved participants with minimal or no resistance training experience. This makes it difficult to generalize our findings to trained individuals. If we consider studies that involved trained participants, both studies (Morton et al., 2016; Cumming et al., 2025) reported negligible fiber hypertrophy throughout the training intervention, indicating a possibility of an attenuated response to training stimulus (Steele et al., 2023).

We did not perform any sex difference analyses as female participants were included only in two studies (Schuenke et al., 2012; Cumming et al., 2025). Therefore, future studies may consider exploring whether there are sex differences in the effects of training load on muscle fiber hypertrophy. Additionally, all biopsy samples were obtained from the vastus lateralis muscle, which limits the generalizability of our findings to other muscle groups. Lastly, variability in the number of muscle fibers and the number of fibers analyzed per participant, as well as the pooling of fiber subtypes, may have influenced our findings.

The pairwise contrasts were derived from model-estimated values across eight load anchors ranging from 20% to 90% 1RM. The observed data spanned only 12 discrete load values between 15.5% and 92.5% 1RM, with a complete absence of observed data between 53% and 70% 1RM within the included studies. Several load anchors included in our contrast analyses (e.g., 20%, 60%, 90% 1RM) therefore represent model estimates with no directly observed data near them. Thus, these estimates should be interpreted as descriptive rather than definitive evidence for a load at which fiber-type specific responses may diverge.

Studies generally reported RM ranges that were used throughout the training intervention. However, exact load values were rarely provided. While a prudent approach was used in our standardized load-estimation procedure, these values remain estimates of the actual training loads, and thus the complete accuracy of this approach is somewhat unclear. Based on our estimates, the majority of studies employed >70% or ≤53% 1RM as high- or low-load, respectively. There is a lack of data available for moderate loads (50-70% 1RM). Therefore, future studies may consider utilizing moderate training loads to investigate whether a load threshold may be apparent when it relates to fiber type-specific hypertrophy.

The review was not prospectively registered. Although the analytical approach was agreed upon by the authors before performing statistical analyses, the lack of a prospectively registered protocol is a limitation. Still, the complete dataset and analysis code have been made publicly available to maximize transparency and reproducibility.

Based on the TESTEX scores, studies had several methodological and reporting limitations. Specifically, studies were commonly downgraded for failing to report eligibility criteria, randomization and allocation concealment procedures, and participant withdrawal and adherence. While these aspects are more a limitation of the included studies, and not this review per se, they should be addressed by future research.

## 6 Conclusions

When both fiber types were combined, the available data were insufficient to determine a meaningful effect of training load on hypertrophy. However, when analyzed separately, our model-derived estimates suggested a possible trend toward a load-specific response. In particular, pairwise contrast estimates were compatible with greater type I hypertrophy at lower loads (20–30% 1RM), whereas the pattern progressively shifted toward relatively greater type II fiber hypertrophy as load increased. These estimates should be considered exploratory and hypothesis-generating given the small number of included studies, methodological and reporting limitations of the included studies, the absence of observed data between 53% and 70% 1RM, and the wide 95% confidence and prediction intervals, indicating a degree of uncertainty and imprecision.

## Data Availability

All extracted data and code utilized for data preparation and analyses are available in the Open Science Framework page for this project (https://osf.io/wgze9/). Other supplementary analyses and plots are also available there.

## Footnotes

1 Where z_0.75_–z_0.25_ is the difference between the 75th and 25th percentiles (~1.3490).
