# Supplementary material for: Heavy or light: is muscle fiber growth load-specific? A systematic review and meta‐regression
Source: Front Physiol. 2026 Oct 6;17:1953601. doi: 10.3389/fphys.2026.1953601 (PMC13680249; doi:10.3389/fphys.2026.1953601)
Supplement: Supplementary file 1 [file SupplementaryFile1.zip › leave_one_out_sensitivity.pdf]

Coefficients for Leave-One-Out Sensitivity Analysis

Load × fiber type interaction estimate with each study sequentially excluded

Study Excluded	β	95% Confidence Interval
Campos et al	0.043	[0.005, 0.081]
Lim et al	0.056	[-0.017, 0.13]
Mitchell et al	0.057	[-0.009, 0.123]
Mackey et al	0.059	[-0.017, 0.134]
Lees et al	0.061	[-0.004, 0.126]
Morton et al	0.062	[-0.028, 0.152]
Schuenke et al	0.062	[-0.014, 0.138]
Cumming et al	0.069	[0.008, 0.13]
