# Supplementary material for: Heavy or light: is muscle fiber growth load-specific? A systematic review and meta‐regression
Source: Front Physiol. 2026 Oct 6;17:1953601. doi: 10.3389/fphys.2026.1953601 (PMC13680249; doi:10.3389/fphys.2026.1953601)
Supplement: Supplementary file 1 [file SupplementaryFile1.zip › Moderator Analyses/moderator_slopes_table.pdf]

Marginal Slopes for Each Interaction Moderator

SMC reflects changes per 10 percentage-point increase in load centered at mean load and averaged over study design

Level	k	n	Fiber Type	SMC	95% Confidence Interval	95% Prediction Interval
Volume Equated						
Equated	3	10	Type II	0.060	[-0.028, 0.149]	[-0.803, 0.923]
Equated	3	7	Type I	0.001	[-0.045, 0.048]	[-0.858, 0.861]
Not Equated	5	12	Type II	0.048	[-0.092, 0.188]	[-0.821, 0.918]
Not Equated	5	10	Type I	-0.011	[-0.151, 0.13]	[-0.88, 0.859]
Study Design						
Between-Subject	4	14	Type II	0.097	[-0.093, 0.287]	[-0.707, 0.901]
Between-Subject	4	9	Type I	0.038	[-0.159, 0.236]	[-0.768, 0.844]
Within-Subject	4	8	Type II	0.028	[-0.054, 0.11]	[-0.758, 0.813]
Within-Subject	4	8	Type I	-0.031	[-0.094, 0.032]	[-0.815, 0.753]
Weekly Set Volume						
High (>20 sets/week)	3	10	Type II	0.076	[-0.076, 0.227]	[-0.806, 0.957]
High (>20 sets/week)	3	6	Type I	0.017	[-0.13, 0.163]	[-0.864, 0.897]
Low (<10 sets/week)	2	4	Type II	0.007	[-0.11, 0.124]	[-0.869, 0.883]
Low (<10 sets/week)	2	4	Type I	-0.052	[-0.142, 0.038]	[-0.925, 0.821]
Medium (10-20 sets/week)	4	8	Type II	0.041	[-0.056, 0.139]	[-0.832, 0.915]
Medium (10-20 sets/week)	4	7	Type I	-0.018	[-0.095, 0.06]	[-0.889, 0.854]
Rest Interval						
Intermediate (90s)	4	10	Type II	0.057	[-0.24, 0.355]	[-1.662, 1.777]
Intermediate (90s)	4	7	Type I	0.011	[-0.272, 0.294]	[-1.706, 1.728]

Marginal Slopes for Each Interaction Moderator

SMC reflects changes per 10 percentage-point increase in load centered at mean load and averaged over study design

Level	k	n	Fiber Type	SMC	95% Confidence Interval	95% Prediction Interval
Long (150s)	1	2	Type II	0.075	[-0.394, 0.545]	[-1.682, 1.833]
Long (150s)	1	1	Type I	0.029	[-0.46, 0.518]	[-1.733, 1.791]
Short (60s)	2	4	Type II	0.048	[-0.221, 0.317]	[-1.666, 1.763]
Short (60s)	2	3	Type I	0.002	[-0.223, 0.227]	[-1.706, 1.71]
Training Status						
Trained	2	4	Type II	0.014	[-0.313, 0.341]	[-2.805, 2.832]
Trained	2	4	Type I	-0.045	[-0.333, 0.244]	[-2.859, 2.77]
Untrained	6	18	Type II	0.072	[-0.242, 0.386]	[-2.745, 2.889]
Untrained	6	13	Type I	0.013	[-0.341, 0.367]	[-2.809, 2.835]
