# Supplementary material for: Heavy or light: is muscle fiber growth load-specific? A systematic review and meta‐regression
Source: Front Physiol. 2026 Oct 6;17:1953601. doi: 10.3389/fphys.2026.1953601 (PMC13680249; doi:10.3389/fphys.2026.1953601)
Supplement: Supplementary file 1 [file SupplementaryFile1.zip › Moderator Analyses/moderator_plots.pdf]

Predicted Marginal Means by Volume Equated Status

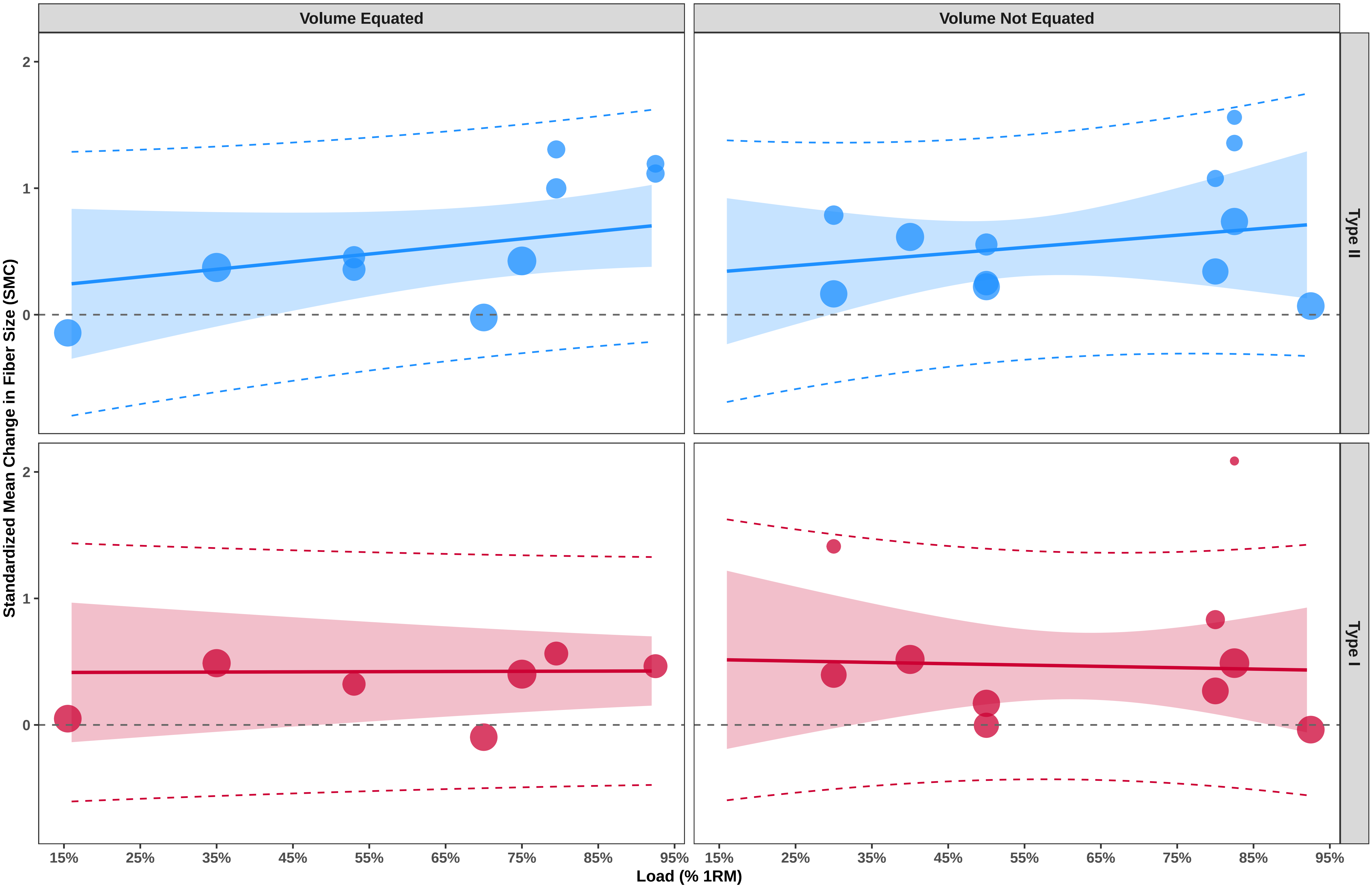

Predicted Marginal Means by Study Design

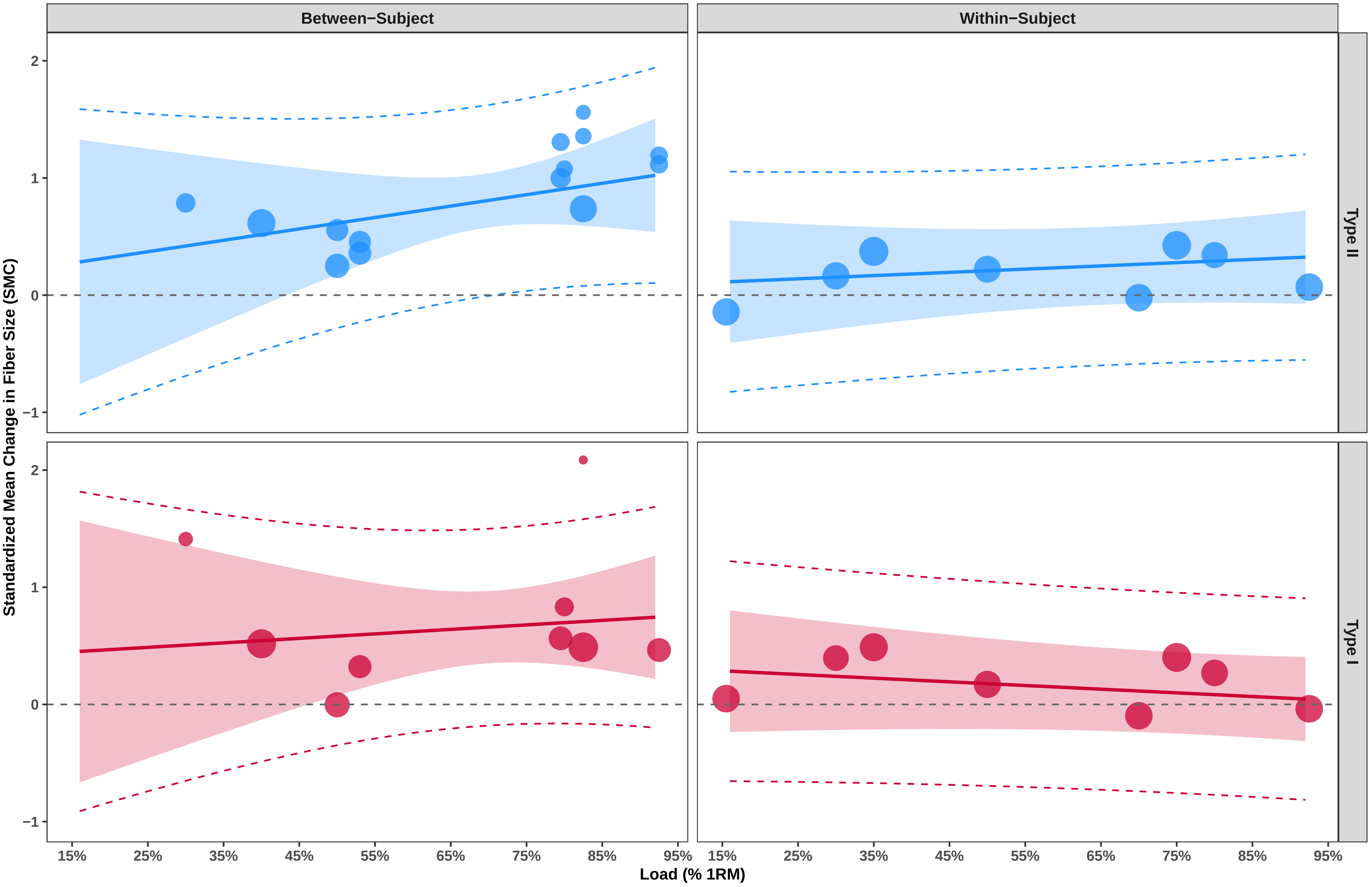

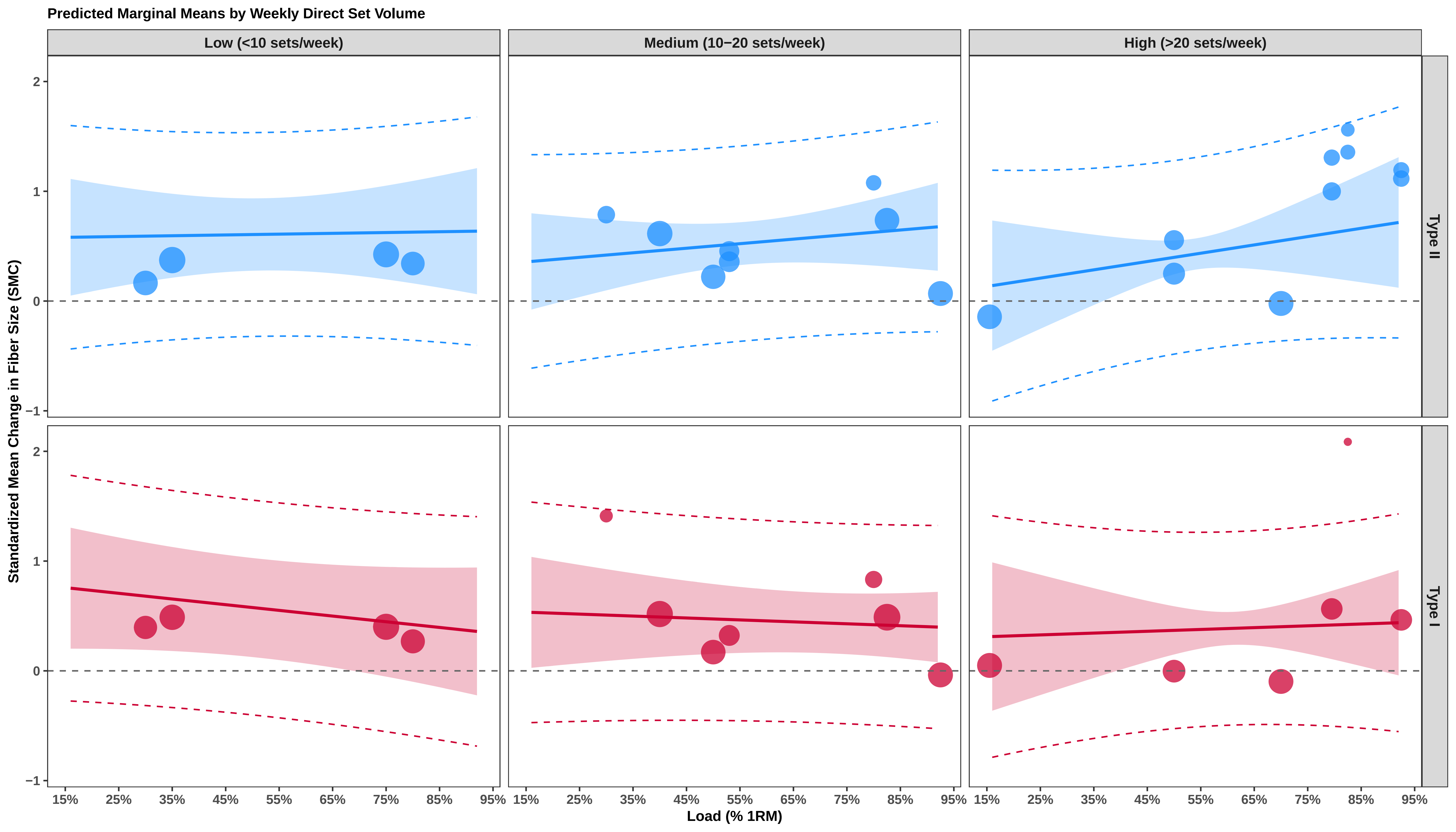
Predicted Marginal Means by Weekly Direct Set Volume
Low (<10 sets/week)
Medium (10–20 sets/week)
High (>20 sets/week)
Type II
Type I
Standardized Mean Change in Fiber Size (SMC)
2
1
0
−1
15%
25%
35%
45%
55%
65%
75%
85%
95%
Load (% 1RM)

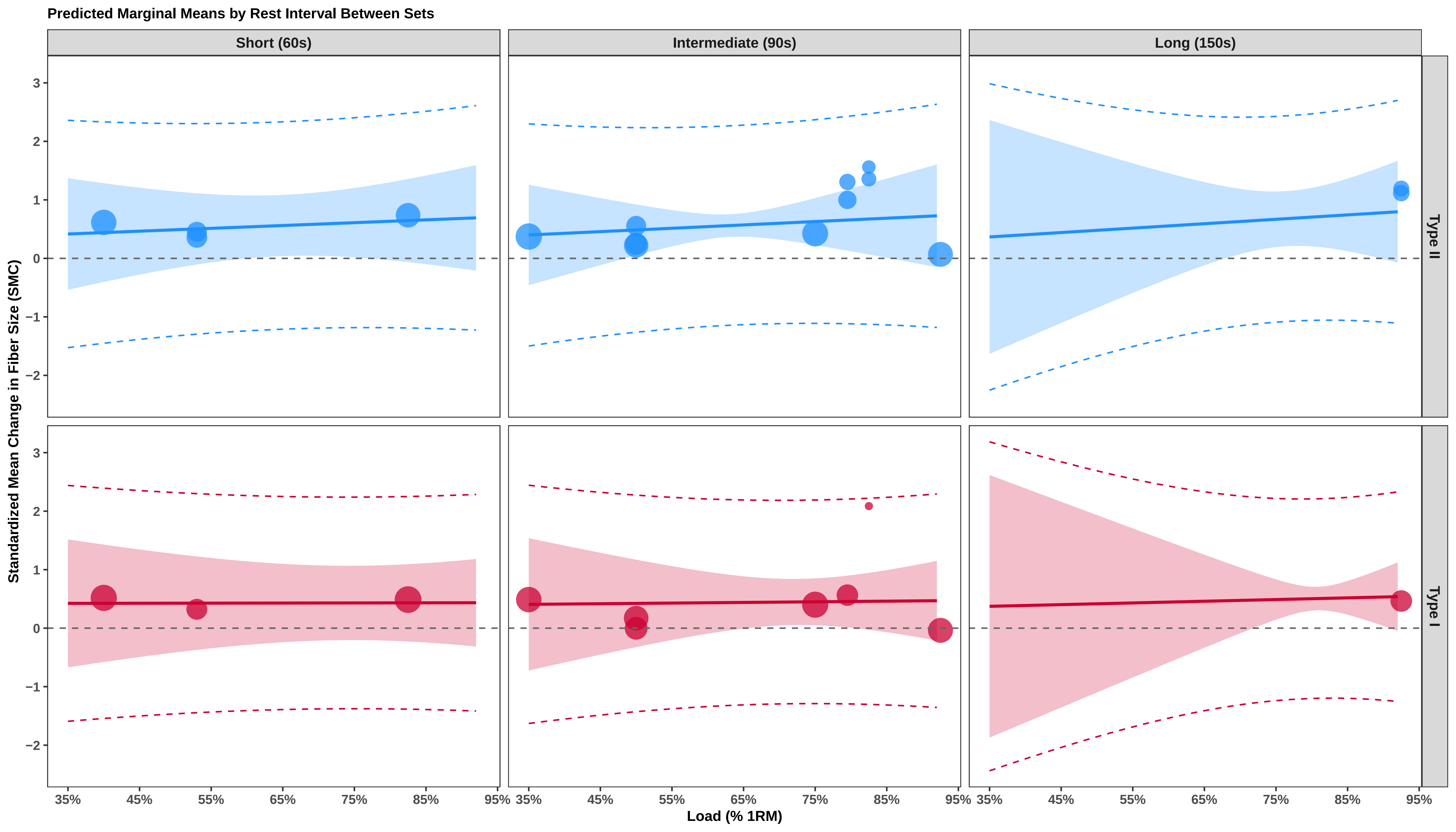

Predicted Marginal Means by Rest Interval Between Sets
Short (60s)
Intermediate (90s)
Long (150s)
Type II
Type I
Standardized Mean Change in Fiber Size (SMC)
Load (% 1RM)
35%
45%
55%
65%
75%
85%
95%
3
2
1
0
−1
−2

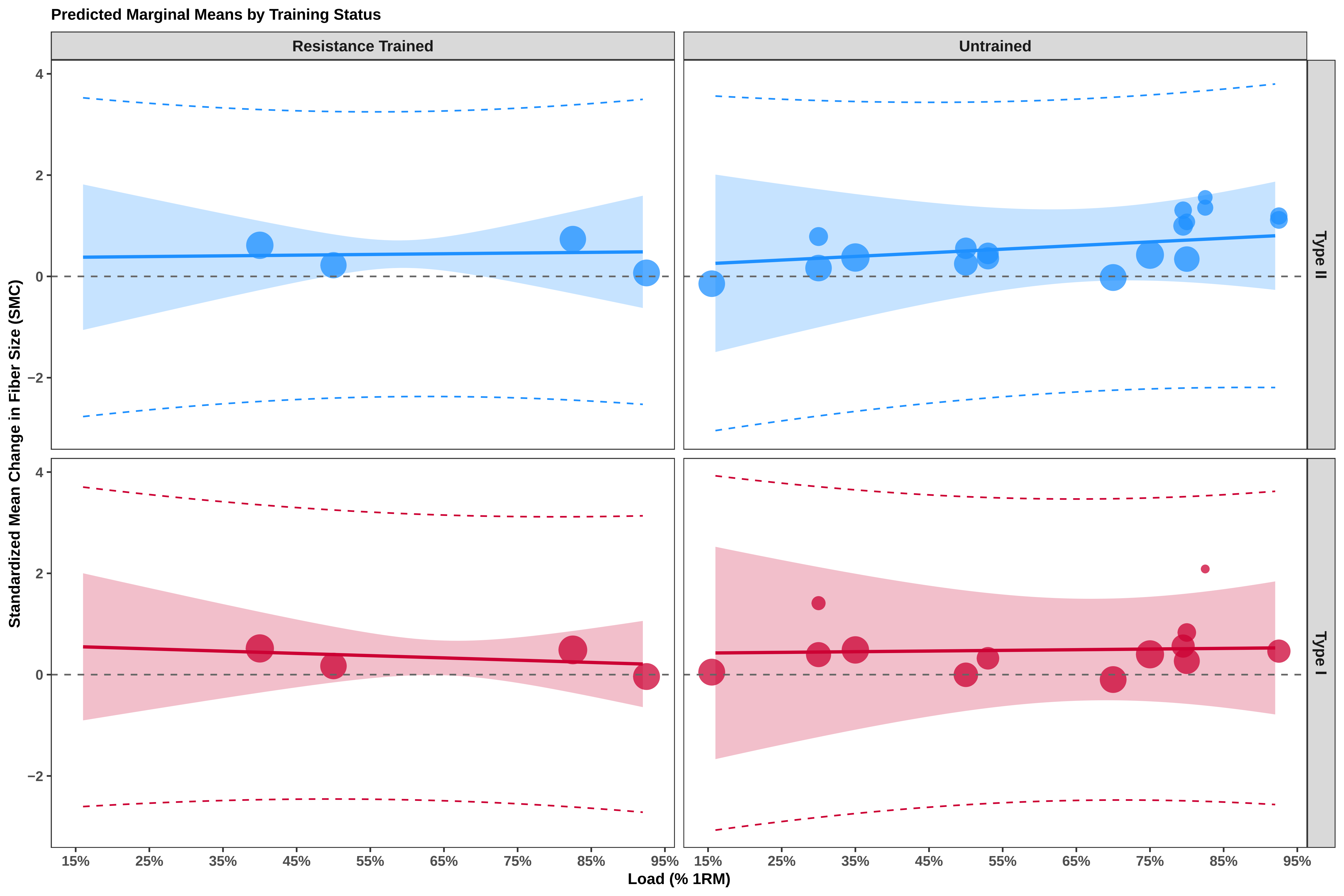

Predicted Marginal Means by Training Status
Resistance Trained
Untrained
Type II
Type I
Standardized Mean Change in Fiber Size (SMC)
Load (% 1RM)
4
2
0
−2
15%
25%
35%
45%
55%
65%
75%
85%
95%
